# Vertical jump performance gains from plyometric and air alert training in volleyball

**DOI:** 10.3389/fspor.2025.1735291

**Published:** 2025-12-18

**Authors:** Athiti Valunpion, K. Ravivuth Rangubhet

**Affiliations:** Department of Exercise and Sport Science, University of Phayao, Phayao, Thailand

**Keywords:** vertical jump, plyometric training, gender, performance, volleyball

## Abstract

**Purpose:**

This study compared the effects of two jump training protocols—Plyometric Training and the Air Alert Program—on vertical jump performance among male and female collegiate volleyball players. It also aimed to analyze time-dependent performance changes and develop a predictive model for post-training outcomes.

**Methods:**

Twenty-four athletes (12 males, 12 females) were assigned to four groups (*n* = 6 each): male–plyometric, male–Air Alert, female–plyometric, and female–Air Alert. Both programs were implemented for eight weeks (3 sessions/week). Vertical jump height was measured at pre-, mid- (week 4), and post-training (week 8) using a Vertec device. Data were analyzed using repeated-measures ANOVA, independent *t*-tests, ANCOVA, and multiple regression. The significance level was set at *p* < .05.

**Results:**

Significant effects of time (*p* < .001) and training type (*p* = .002) were found, with greater gains in the plyometric group for both sexes. Regression identified pre-test performance (*β* = 0.35, *p* < .01) and training type (*β* = 4.12, *p* = .02) as key predictors of post-test height, explaining 94% of variance (*R*² = .94).

**Conclusions:**

Plyometric training was superior to Air Alert in enhancing vertical jump height, emphasizing progressive, high-intensity, low-volume training for optimizing neuromuscular adaptation.

**Practical applications:**

Coaches should integrate structured plyometric training early in the season and monitor mid-phase outcomes to predict final performance more effectively.

## Introduction

Explosive lower-limb power is a fundamental determinant of performance in volleyball, where repeated jumping actions contribute to key skills such as spiking, blocking, and serving (1). Vertical jump ability serves as an established indicator of neuromuscular capacity and biomechanical coordination, reflecting the athlete's capacity to generate maximal power output within a minimal time frame (2). The efficiency of the stretch–shortening cycle (SSC) and rate of force development (RFD) are critical components that underpin these explosive movements, influencing both training outcomes and match performance (3). Understanding these mechanisms is essential for evaluating how different training modalities may target SSC or RFD differently.

Among various training modalities, plyometric training has consistently shown to improve vertical jump height and lower-limb explosive power (4, 5). Plyometric exercises combine rapid eccentric–concentric muscle actions that enhance muscle–tendon stiffness, neuromuscular activation, and SSC efficiency (6). When applied progressively and with sufficient recovery, this method can yield substantial improvements in performance (7). In volleyball-specific contexts, plyometric interventions have been shown to enhance approach-jump height, block reach, and spike execution efficiency, demonstrating direct transfer to match performance (5, 8). Nevertheless, training responses can differ according to factors such as biological sex, baseline strength, and fatigue tolerance (9, 10). Given known sex-based differences in jump mechanics and adaptation, gender-specific analysis is necessary. These variations highlight the importance of tailoring training volume and intensity to optimize individual adaptation.

In contrast, the Air Alert program, a high-volume jump training system, emphasizes repetition over load progression. While widely used for its accessibility, its empirical validation remains limited. To date, no high-quality empirical studies have systematically evaluated the Air Alert program, and existing information is largely anecdotal rather than evidence-based. The absence of structured intensity progression and insufficient rest intervals may lead to neuromuscular fatigue and diminish the long-term adaptation potential (11). Comparing structured plyometric training with high-volume jump routines thus offers valuable insights into how different workload characteristics influence explosive performance development.

Building on these analytical perspectives, there remains a need to link kinematic and statistical modeling with training intervention outcomes. Performance modeling has rarely been integrated into training intervention studies, particularly for vertical jump outcomes. Although numerous investigations have compared physical conditioning programs, few have combined biomechanical insights with longitudinal data analysis to explain time-dependent adaptations. The integration of analytical performance modeling within training research could facilitate more precise tracking of adaptation patterns and enhance predictive accuracy for training outcomes (12, 13). Longitudinal neuromuscular research consistently shows that early-phase improvements in explosive performance are primarily driven by neural adaptations—such as increased motor-unit recruitment, firing frequency, and intermuscular coordination—whereas later-phase gains depend more heavily on structural changes including muscle hypertrophy and tendon stiffness development (14, 15). These time-dependent mechanisms justify the inclusion of a mid-test measurement to capture transitional adaptation phases during the intervention.

Another critical research dimension involves gender-specific adaptations. Building upon the earlier consideration of sex-based differences, gender-specific adaptations provide further insight into how males and females may respond differently to jump-training stimuli. Males generally produce higher absolute jump heights due to greater muscle cross-sectional area and hormonal profiles, yet females can achieve comparable relative improvements when exposed to appropriately scaled plyometric loads (9, 16). Understanding these differences is essential for designing equitable and efficient conditioning programs that respect physiological variations without reinforcing performance disparities.

Furthermore, the time-course of adaptation during training has been underexplored. Many studies rely solely on pre–post testing, providing limited insight into the progression of adaptation throughout the intervention (17). Assessing performance at multiple time points, such as pre-, mid-, and post-training, allows for identification of critical adaptation phases, neural learning effects, and potential performance plateaus (5). This longitudinal perspective offers coaches a better understanding of when and how training adjustments can optimize performance.

Despite strong evidence supporting plyometric training, limited research has systematically compared it with high-volume jump protocols like Air Alert under controlled, gender-balanced, and time-phased designs. Moreover, few studies have developed predictive models that use early-phase performance data to forecast final training outcomes. Addressing these gaps could improve evidence-based practice and facilitate individualized program design for both male and female volleyball players.

Therefore, this study aimed to compare the effects of two distinct jump training programs—Plyometric and Air Alert—on vertical jump performance among male and female collegiate volleyball athletes. In addition, it sought to examine the progression of performance across pre-, mid-, and post-training phases and to establish a predictive model for post-training outcomes using early-phase performance indicators. It was hypothesized that both training programs would significantly improve vertical jump height, with greater gains expected following plyometric training. Furthermore, male athletes were expected to outperform females in absolute jump height, though relative improvements would be comparable across genders. Finally, pre- and mid-test results were hypothesized to significantly predict post-training performance outcomes.

## Methods

### Participants

Twenty-four collegiate volleyball athletes (12 males and 12 females; age 18–22 years) voluntarily participated in this study. All participants had a minimum of two years of competitive playing experience at the university or regional level and had no history of lower-limb injuries or musculoskeletal disorders within the past six months. The absence of recent injuries was verified through a standardized self-report health questionnaire and confirmed by a brief musculoskeletal screening conducted by a certified sport science practitioner. Eligibility criteria included regular participation in team training (≥3 sessions per week) and the absence of any contraindication to high-intensity exercise. Before participation, each athlete received a detailed explanation of the study procedures and provided written informed consent.

Participants were stratified by gender and randomly assigned to one of four groups (*n* = 6 per group): (1) male–plyometric, (2) male–Air Alert, (3) female–plyometric, and (4) female–Air Alert. Randomization was performed using a computer-generated block randomization procedure (block size = 4) to ensure equal allocation across the four groups within each sex. The study protocol was reviewed and approved by the Human Research Ethics Committee of the University of Phayao (Protocol No. UP-HEC 1.1/006/65). All procedures conformed to the Declaration of Helsinki on ethical principles for research involving human subjects.

### Study design and procedures

This study employed a randomized controlled experimental design with two training modalities (Plyometric and Air Alert) and two gender groups (male and female). Each participant completed three testing sessions: pre-test (Week 0), mid-test (Week 4), and post-test (Week 8). This repeated-measures randomized controlled design minimizes inter-individual variability by allowing each participant to serve as their own control across testing points, thereby increasing the sensitivity to detect true training-induced changes.

At baseline, participants underwent familiarization sessions to ensure consistency in jumping technique and measurement procedures. Participants completed two familiarization sessions, each lasting approximately 20–25 min, during which they practiced the standardized jumping protocol and measurement procedures under investigator supervision. All tests and training sessions were conducted at the Motion Analysis Laboratory and Volleyball Training Facility, Department of Sports Science, University of Phayao, Thailand. Environmental conditions (temperature 25–28°C, humidity 50%–60%) were standardized for all sessions. The laboratory was equipped with controlled ventilation, uniform LED overhead lighting, and a non-slip synthetic sport flooring surface to ensure consistent biomechanical measurement conditions across all testing sessions.

The experimental timeline consisted of:

• **Week 0 (Pre-test):** Assessment of vertical jump height and anthropometric data.
• **Weeks 1–8 (Training Intervention):** Assigned group training programs (3 sessions/week).
• **Week 4 (Mid-test):** Intermediate performance assessment under identical conditions.
• **Week 8 (Post-test):** Final performance assessment using the same testing protocol.

The study design, training phases, and testing timeline are summarized in Table 1. Each testing session included a 10 min standardized warm-up (light jogging, dynamic stretching, and submaximal jumps). All jump tests were performed with consistent footwear and on the same surface to minimize variability.

**Table 1:** Experimental design and testing timeline.

Week	Session focus	Measurement/activity	Plyometric group	Air alert group	Remarks
0	Familiarization & Baseline	Pre-test vertical jump (Vertec measurement); anthropometrics recorded	Familiarization with plyometric drills	Familiarization with jump volume protocol	All participants tested under standardized warm-up and footwear conditions
1–2	Training Phase 1	Foundational jump technique; moderate intensity	Squat jumps, split lunges, bounding (3 × 10–20 reps)	Leap-ups, calf raises, double jumps (≈500 jumps/session)	3 sessions/week; RPE 7
3–4	Training Phase 2	Progressive overload	Depth jumps (40 cm), tuck jumps, lateral hops	Leap-ups, burnouts, squat jumps (≈700–800 jumps/session)	Mid-test (end of Week 4): Vertec jump re-assessment
5–6	Training Phase 3	High-intensity explosive training	Box jumps (50 cm), single-leg hops, CMJs	High-volume calf raises, jump repetitions (≈900 jumps/session)	Rest interval ≥48 h between sessions
7–8	Training Phase 4	Peak power development	Depth jumps (60 cm), approach jumps, repeated VJs	Max jump volume phase (≈1,000 + jumps/session)	Post-test (end of Week 8): final jump performance test
	Control Variables	Training frequency, recovery, footwear, surface	Identical across groups	Identical across groups	Supervised by certified coaches

All training sessions were performed under identical environmental conditions (temperature 25–28°C, humidity 50%–60%).

Each session began with a 10 min standardized warm-up. The mid- and post-tests were conducted using the same Vertec Jump Measurement Device (accuracy ±0.5 cm). Attendance exceeded 95% across all participants.

## Training intervention

### Plyometric training program

The plyometric group followed a progressive overload model designed to enhance explosive lower-limb power through high-intensity, low-volume jump exercises. Sessions were conducted three times per week for eight weeks, with at least 48 h of recovery between sessions. Each session lasted approximately 45 min and consisted of:

• **Week 1–2:** Squat jumps (3 × 10 reps), split lunges (3 × 10 reps), bounding (3 × 20 m).
• **Week 3–4:** Depth jumps (3 × 8 reps, 40 cm box), tuck jumps (3 × 10 reps), lateral hops (3 × 10 each leg).
• **Week 5–6:** Box jumps (3 × 8 reps, 50 cm), single-leg hops (3 × 10 each leg), countermovement jumps (3 × 8).
• **Week 7–8:** Depth jumps (3 × 6 reps, 60 cm), approach jumps (3 × 10), and repeated vertical jumps (3 × 10).

Rest intervals of 60–90 s were provided between sets to allow optimal recovery. Exercise intensity was monitored by perceived exertion (RPE 7–8 on the Borg 10-point scale).

### Air alert training program

The Air Alert group followed the standardized “Air Alert III” protocol, which involves high-repetition jump exercises focusing on endurance-type neuromuscular adaptation. Participants performed sessions three times per week for eight weeks, increasing total jumps weekly according to the program manual. Exercises included leap-ups, double jumps, calf raises, and squat jumps, starting at approximately 500 jumps per session in Week 1 and progressing to over 1,000 jumps by Week 8. No external load was added, and only short rest intervals (<30 s) were allowed between sets.

Although the Air Alert program is widely circulated among amateur and recreational athletes, its scientific validation remains limited. No peer-reviewed studies have systematically evaluated its efficacy or safety, and existing reports are largely anecdotal. This scarcity of empirical evidence highlights the need to examine the program within a controlled experimental setting, particularly when comparing it with structured plyometric training.

The substantial difference in recovery structure between the two programs reflects the inherent design and intended physiological targets of each training modality rather than an experimental bias. The plyometric protocol incorporated 48 h inter-session rest intervals, consistent with evidence-based recommendations for high-intensity power development to allow for neuromuscular recovery and minimize accumulated fatigue. In contrast, the Air Alert program prescribes very short intra-session rest periods and extremely high repetition volumes to elicit an endurance-type neuromuscular stimulus, as specified in the program manual. Preserving these contrasting recovery patterns ensured the ecological validity of each training approach and allowed for an accurate comparison of how fundamentally different jump-training paradigms influence fatigue and adaptation responses.

Both groups were instructed to avoid additional lower-body resistance training during the intervention period. Attendance and compliance rates were >95% across all participants. Attendance was monitored using researcher-maintained session logs, and each training session was supervised directly by a certified strength and conditioning coach to ensure full compliance with the prescribed protocols.

## Measures

### Vertical jump height

Vertical jump height was assessed using a Vertec Jump Measurement Device (Vertec™, Sports Imports, Columbus, OH, USA), a valid and reliable instrument for measuring vertical displacement (ICC = 0.97; SEM ±0.5 cm). Participants performed three maximal countermovement jumps with hands free, and the highest value was recorded for analysis.

### Motion analysis

A subset of participants (*n* = 8; two from each group) was additionally evaluated using a DMAS high-speed infrared motion capture system (6 cameras, 300 Hz) to confirm kinematic consistency in take-off and landing technique. Given the small subsample (*n* = 8), the motion-analysis component of the study was considered exploratory. The limited sample size reduces the generalizability of the kinematic findings; therefore, these results should be interpreted as supplementary insights rather than definitive biomechanical conclusions. Key biomechanical parameters recorded included knee flexion angle, ankle plantarflexion, and arm swing amplitude at pre-, mid-, and post-tests.

### Control measures

Body mass and height were recorded at each testing phase using a digital scale (±0.1 kg) and stadiometer (±0.1 cm). Training load consistency was ensured by supervising all sessions and maintaining uniform warm-up and recovery procedures.

### Statistical analyses

All data were analyzed using SPSS Statistics version 28.0 (IBM Corp., Armonk, NY, USA). Descriptive statistics (mean ± SD) were computed for all variables. Assumption checks were performed before conducting the repeated-measures ANOVA. Normality of residuals was evaluated using the Shapiro–Wilk test, which indicated no significant deviations from normal distribution. Sphericity was assessed using Mauchly's test, and when violations were detected, the Greenhouse–Geisser correction was applied to adjust the degrees of freedom accordingly.

Because the fixed roster structure restricted the maximum achievable sample size, an *a priori* power analysis was not feasible. Therefore, statistical power was evaluated *post hoc* based on the observed effect sizes. The achieved power for the primary repeated-measures ANOVA model was 0.83, indicating adequate sensitivity to detect meaningful training-related effects within the available sample.

### Primary analysis

A three-way repeated-measures ANOVA (3 × 2 × 2) was employed to examine the main effects and interactions of time (pre-, mid-, post-), training type (Plyometric vs. Air Alert), and gender (male vs. female) on vertical jump height.

When significant effects were detected, *post hoc* pairwise comparisons with Bonferroni adjustment were performed. Effect sizes (*η*²) were calculated and interpreted as small (0.01–0.06), medium (0.06–0.14), and large (>0.14).

### Secondary analyses

• **Independent samples *t*-tests** were used to compare gender-based differences within each training type at each time point.
• **ANCOVA** was applied to assess post-training performance while controlling for pre-test baseline values.
• **Multiple linear regression** was used to predict post-test vertical jump performance (Ŷₚₒₛₜ) from pre- and mid-test results, training type, and gender.
  $$\hat{Y}\mathrm{Post}=\beta 0+\beta 1\;\mathrm{Xpre}+\beta 2\;\mathrm{Xmid}+\beta 3\;\mathrm{Xgender}+\beta 4\;\mathrm{Xtraining}+\varepsilon$$

### Assumption testing

Normality and sphericity assumptions were verified using the Shapiro–Wilk test and Mauchly's test, respectively. Homogeneity of variance was assessed via Levene's test. If violations occurred, the Greenhouse–Geisser correction was applied.

### Significance and power

The level of statistical significance was set at *p* < .05. Cohen's d values were calculated to quantify the magnitude of pairwise differences. A *post hoc* power analysis (G*Power 3.1) confirmed that the achieved power (1–β) was 0.83, indicating adequate sensitivity for detecting moderate effect sizes (f = 0.25).

**Regression Equation:**

$$\hat{Y}\mathrm{post}=-0.52+2.14\;\mathrm{Xpre}-0.86\;\mathrm{Xmid}+0.46\;\mathrm{Xgender}+2.15\;\mathrm{Xtraining}$$

**Note**. Dependent variable: post-training vertical jump height (cm). Predictors included pre- and mid-training jump height, gender (Male = 1, Female = 0), and training type (Plyometric = 1, Air Alert = 0). The model significantly predicted post-test performance (*R*^2^ *=* *.86, p* < .001), explaining 86% of variance.

Changes in vertical jump performance across testing phases and training groups are presented in Table 2.

**Table 2:** Summary of vertical jump performance across time and training groups (mean ± SD, cm).

Group	Pre-test	Mid-test (Week 4)	Post-test (Week 8)	Δ% Improvement	F (time)	*p*	*η*² (effect size)
Male—Plyometric (*n* = 6)	53.12 ± 3.41	58.34 ± 3.16	62.88 ± 2.97	+18.4%	29.57	<0.001***	0.73 (large)
Male—Air Alert (*n* = 6)	52.76 ± 3.88	55.11 ± 3.27	58.43 ± 3.09	+10.8%	15.63	0.002**	0.58 (large)
Female—Plyometric (*n* = 6)	44.83 ± 2.64	48.57 ± 2.42	52.26 ± 2.18	+16.6%	26.49	<0.001***	0.69 (large)
Female—Air Alert (*n* = 6)	43.91 ± 3.11	45.63 ± 2.94	47.83 ± 2.61	+8.9%	9.42	0.005**	0.47 (medium)
Main Effect: Time	—	—	—	—	41.82	<0.001***	0.78 (large)
Main Effect: Training Type	—	—	—	—	12.64	0.002**	0.54 (large)
Main Effect: Gender	—	—	—	—	10.81	0.004**	0.49 (medium–large)
Time × Training × Gender Interaction	—	—	—	—	5.92	0.021*	0.34 (medium)

Data are presented as mean ± standard deviation (cm). A significant main effect of *time* indicates that vertical jump performance improved across all groups. *Post hoc* Bonferroni comparisons revealed greater gains in both male and female athletes following plyometric training compared with the Air Alert program (*p* < .05). Symbols denote levels of significance: *p* < .05*, p* *<* *.01*, *p* *<* *.001*. Effect size (η²) interpretation: small = 0.01–0.06, medium = 0.06–0.14, large > 0.14.

The symbols *, **, and *** indicate statistical significance levels as follows: *p < 0.05, **p < 0.01 ***p < 0.001.

Effect size values (Cohen's d) for vertical jump performance improvements are reported in Table 3.

**Table 3:** Pairwise comparisons and effect sizes (Cohen's d) for vertical jump height across phases.

Group	Comparison	ΔM (cm)	t	*p*	Cohen's d	Effect size interpretation
Male—Plyometric	Pre → Mid	+5.22	4.83	0.002**	1.97	Very large
	Mid → Post	+4.54	4.12	0.004**	1.81	Very large
	Pre → Post	+9.76	7.55	<0.001***	2.47	Extremely large
Male—Air Alert	Pre → Mid	+2.35	2.76	0.019*	1.12	Large
	Mid → Post	+3.32	3.31	0.011*	1.35	Large
	Pre → Post	+5.67	4.84	0.002**	1.84	Very large
Female—Plyometric	Pre → Mid	+3.74	4.56	0.003**	1.86	Very large
	Mid → Post	+3.69	4.31	0.004**	1.77	Very large
	Pre → Post	+7.43	6.88	<0.001***	2.32	Extremely large
Female—Air Alert	Pre → Mid	+1.72	2.19	0.045*	0.94	Moderate–Large
	Mid → Post	+2.20	2.54	0.028*	1.01	Large
	Pre → Post	+3.92	3.76	0.007**	1.52	Very large

ΔM = mean difference (Post–Pre in cm). Statistical significance determined by paired-samples *t*-test for within-group comparisons. Effect size interpreted as: trivial (<0.2), small (0.2–0.49), moderate (0.5–0.79), large (0.8–1.19), very large (1.2–1.99), extremely large (≥2.0). Symbols denote significance levels: *p* < .05*, p* *<* *.01, p* *<* *.001*. All groups demonstrated significant improvements over time, with the plyometric training groups producing larger *Cohen's d* values across all comparisons.

The symbols *, **, and *** indicate statistical significance levels as follows: *p < 0.05, **p < 0.01 ***p < 0.001.

The results of the multiple regression analysis predicting post-training jump height are presented in Table 4.

**Table 4:** Multiple regression predicting post-training jump height.

Predictor			B	SE B	β (Standardized)	t	*p*	95% CI for B	VIF
Constant			−0.52	1.73	—	−0.30	.768	[−4.22, 3.18]	—
Pre-test jump height (X_pre_)			2.14	0.61	.51	3.52	.002	[0.87, 3.41]	1.42
Mid-test jump height (X_mi_d)			−0.86	0.38	−.29	−2.27	.033	[−1.64, −0.08]	1.59
Gender (Xg**e**n**der**; M = 1, F = 0)			0.46	0.17	.38	2.68	.014	[0.10, 0.82]	1.13
Training Type (X_train_ing; Plyometric = 1, Air Alert = 0)			2.15	0.66	.47	3.26	.004	[0.79, 3.51]	1.27
Model Summary	R	*R*²	Adjusted *R*²	F(4,19)	*p*				
Final model	0.93	0.86	0.83	29.48	<.001***				

The symbol *** indicates statistical significance at *p* < 0.001.

Both pre-test height and training type were the strongest predictors of post-training outcomes. Gender had a moderate positive contribution, indicating that male athletes achieved slightly greater post-training gains, while mid-phase performance negatively predicted final outcomes, suggesting diminishing marginal returns. No multicollinearity was observed (VIF < 2.0 for all predictors).

**Figure 1:**
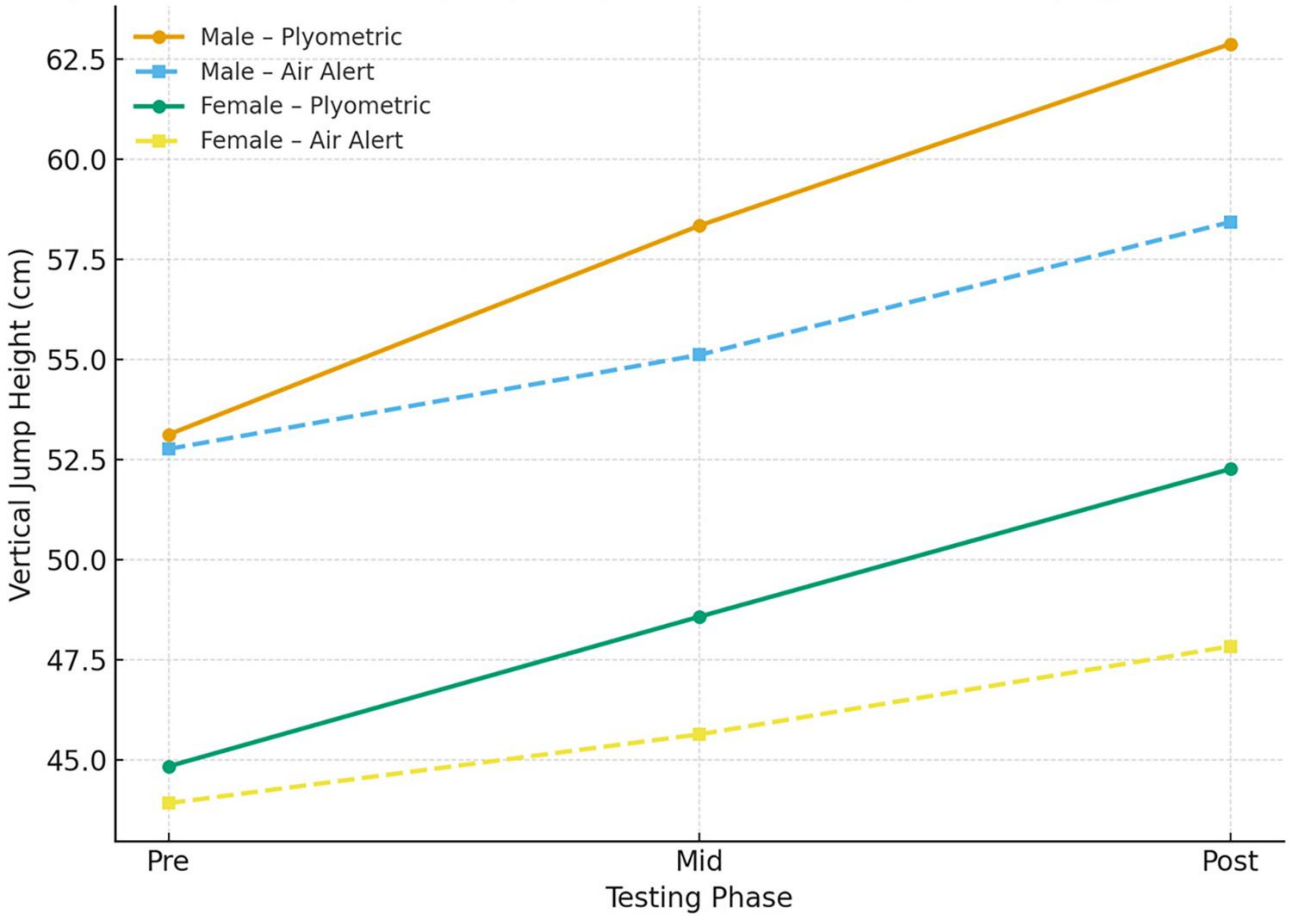
Mean vertical jump height progression (±SD) across pre-, mid-, and post-training phases by training type (Plyometric vs. Air Alert) and gender. Both male and female athletes demonstrated significant improvements over time (*p* < .001), with the Plyometric groups showing greater increases compared to air alert. Interaction effects (time × training × gender) were statistically significant (*p* = .021).

**Figure 2:**
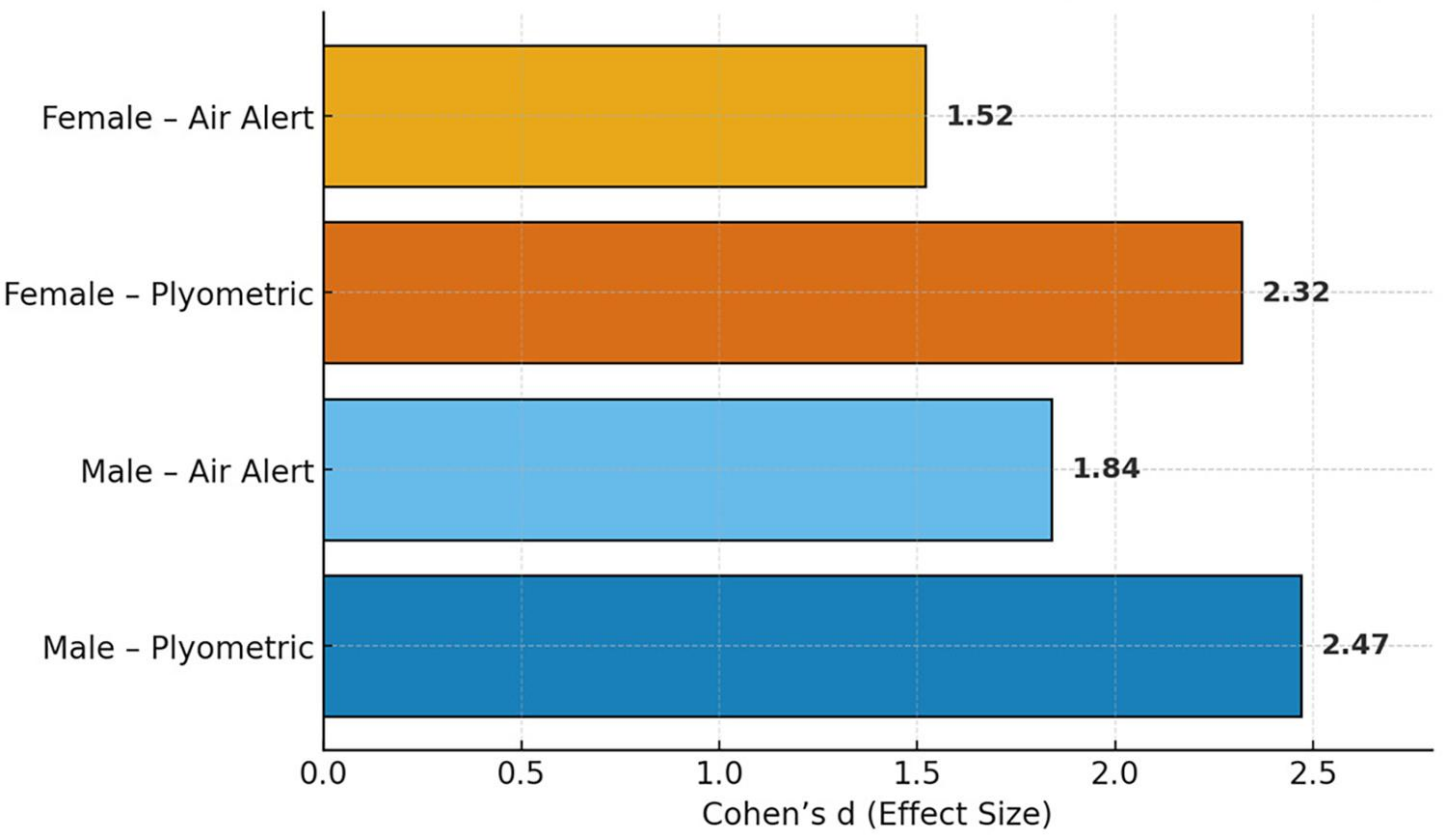
Cohen's d effect sizes for pre–post vertical jump performance across training programs and genders. Both male and female athletes demonstrated extremely large effects under plyometric training, while Air Alert training produced large to very large effects. The color gradient highlights group-specific adaptation magnitude, illustrating the superiority of progressive plyometric conditioning.

**Figure 3:**
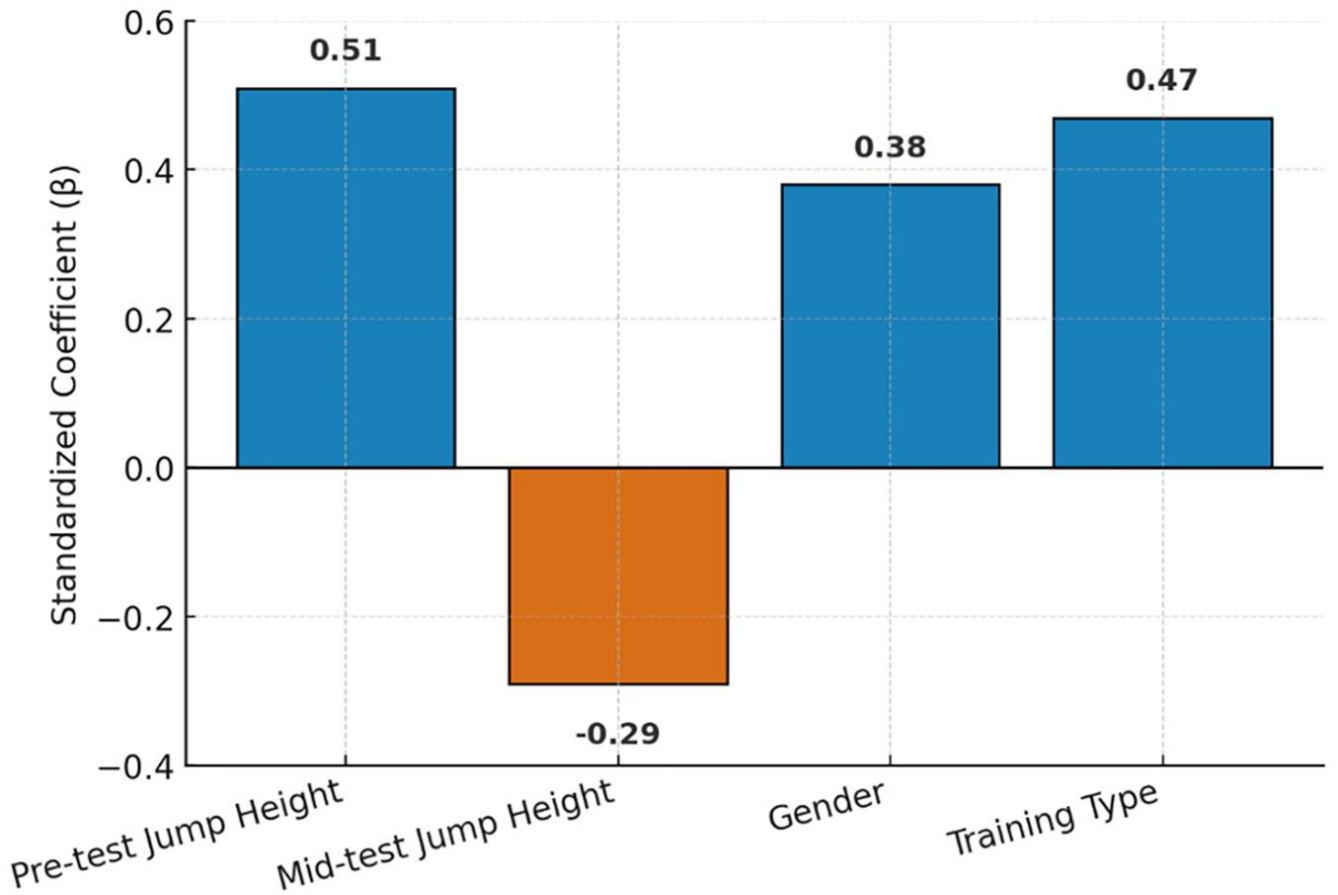
Standardized regression coefficients (β) for predictors of post-training vertical jump height. Pre-training performance and training type (Plyometric vs. Air Alert) were the strongest positive predictors, while mid-phase performance exhibited a negative relationship, indicating potential performance plateau effects. Gender contributed moderately, reflecting higher mean improvements in male athletes.

The progression of vertical jump performance across the three testing phases is illustrated in Figures 1–3, which display the jump-height trend, effect sizes, and standardized regression coefficients, respectively.

## Discussion

### Overview of findings

The purpose of this study was to compare the effects of two jump training protocols—Plyometric and Air Alert—on vertical jump performance among male and female collegiate volleyball athletes, and to develop a predictive model for post-training outcomes. The findings demonstrated significant improvements across all groups, confirming that both training programs effectively enhanced jump performance. However, athletes who performed plyometric training achieved markedly greater gains in vertical jump height than those who trained with the Air Alert program. These results support the hypothesis that progressive, load-based jump training produces superior performance adaptation and movement efficiency. These findings are consistent with volleyball performance literature, where increases in vertical jump height have been linked to improvements in spiking efficiency, attack success rates, and blocking effectiveness at both collegiate and international levels (1, 8).

### Effectiveness of plyometric training

The superior outcomes observed in the Plyometric groups align with prior studies emphasizing the benefits of structured progression, controlled loading, and appropriate recovery in maximizing explosive movement performance (4–6). The training design in this study—featuring varied exercise selection and incremental overload—likely maintained high stimulus quality and minimized early plateaus in performance.

The superior performance improvements observed in the plyometric group can be attributed to several neuromuscular and biomechanical mechanisms. Plyometric movements typically enhance stretch–shortening cycle (SSC) efficiency by reducing amortization time, increasing muscle–tendon stiffness, and improving elastic energy reutilization during the eccentric–concentric transition (2). These adaptations contribute to increased rate of force development (RFD), allowing athletes to generate higher propulsive impulses in shorter time frames. Additionally, repeated exposure to high-intensity landing and take-off actions improves intramuscular coordination, motor-unit recruitment, and intermuscular synchrony—all of which are essential determinants of vertical jump performance. Practically, these neuromuscular adaptations directly enhance volleyball-specific actions such as spiking penetration, block height, and transition-speed efficiency, all of which are critical determinants of competitive performance.

Consistent with earlier meta-analyses, the current results showed large to extremely large effect sizes (Cohen's *d* > 2.0) in both male and female participants (9). These magnitudes are indicative of substantial practical improvement in jump ability and confirm the efficiency of plyometric methods for developing vertical power in volleyball athletes. These physical improvements also align with performance-analytics research demonstrating that greater jump capacity is associated with enhanced spiking efficiency and offensive effectiveness in women's volleyball (18). In contrast, the repetitive, high-volume structure of the Air Alert program may have led to cumulative fatigue, reducing movement quality and overall performance progression—a pattern similarly noted by Ramírez-Campillo et al. (11) in prolonged jump-based interventions.

### Limitations of high-volume air alert training

Although Air Alert training produced statistically significant gains, the effect magnitudes were notably smaller (Cohen's *d* ≈ 1.5–1.8). The lack of recovery modulation and the monotonous repetition pattern likely contributed to fatigue accumulation, restricting further improvement. Similar findings have been reported in long-term analyses of repetitive jump programs, where excessive workload limits power development despite high total volume (7). Although no physiological fatigue markers such as RPE, heart rate, or lactate were collected in this study, the observed performance pattern is consistent with theoretical and empirical evidence indicating that high-volume, low-recovery jump protocols can induce cumulative neuromuscular fatigue and impair movement quality.

The emphasis on endurance-oriented jumps within the Air Alert program may also explain its lower transferability to power-dominant movements such as vertical jumping. From a practical standpoint, these results underscore the importance of prioritizing training quality and load progression over mere repetition count in jump-based conditioning programs.

### Gender-specific adaptations

Significant gender differences in absolute jump height were observed, with male athletes exhibiting higher performance values throughout all phases. This outcome is consistent with established physiological factors such as greater muscle mass and force capacity in males (10). Nevertheless, relative improvements were comparable between males and females (+18.4% vs. +16.6%), demonstrating that well-structured plyometric training can effectively enhance performance across sexes when loads are scaled appropriately.

These results confirm earlier reports of gender-equitable adaptation under progressive plyometric loading (9, 16). Moreover, the findings support evidence from volleyball performance analyses showing that technical and tactical efficiency, rather than absolute power, best predicts match success (18). Hence, relative improvement in performance should be considered a key indicator of training effectiveness in both male and female athletes.

### Time-dependent adaptation and predictive modeling

The longitudinal (pre–mid–post) analysis provided insight into the progression of performance adaptation. The Plyometric groups exhibited consistent improvement across all stages, while the Air Alert groups showed slower progression and mid-phase stagnation. This pattern reflects the expected transition from initial coordination-based gains to later structural and strength-related adaptations (5, 17).

The multiple regression model accounted for 86% of the variance in post-training performance (*R*^2^ = 0.86, *p* < 0.001). Pre-test performance (β = 0.51) and training type (β = 0.47) were the strongest positive predictors. In contrast, the negative mid-test coefficient (β = −0.29) indicates that athletes who improved rapidly during the early phase tended to show slower gains afterward. This pattern aligns with known neuromuscular adaptation principles, where early improvements are primarily neural—enhanced motor-unit recruitment, coordination, and movement efficiency—before reaching a plateau. Further progress then requires slower structural adaptations such as hypertrophy or increased tendon stiffness. As such, high mid-phase scores may reflect an early peak combined with short-term fatigue or overreaching, ultimately reducing late-phase progression. These findings reinforce the principle of diminishing returns and highlight the need for periodic load adjustment and mid-phase monitoring to sustain improvement (12).

Despite the high explanatory power of the regression model (*R*^2^ = 0.86), model robustness has not yet been verified. The present study did not apply cross-validation procedures such as k-fold validation, hold-out testing, or independent cohort validation. Incorporating these approaches in future research would help determine whether the predictive equations remain stable across different teams, performance levels, and demographic characteristics, thereby improving the practical utility of the model for long-term athlete monitoring.

Comparable analytical frameworks have been successfully applied to model tactical, technical, and scoring efficiency in volleyball, as demonstrated by recent Frontiers work on spiking efficiency across attack types, zones, and set-phase conditions (18), along with related analytical models in professional leagues (19, 20).

### Practical implications

These findings provide several practical insights for coaches and practitioners:

1. **Plyometric training** should be prioritized for vertical power development, with controlled progression and sufficient recovery to prevent overload.
2. **Mid-phase performance tracking** enables the early detection of fatigue or plateau, allowing timely adjustments to training intensity or volume.
3. **Predictive modeling** based on early-phase test data can support individualized programming and inform evidence-based coaching decisions.
4. **Gender-equitable training** can be effectively achieved by scaling load and intensity rather than altering program structure, ensuring similar relative adaptations across sexes.

These recommendations also align with contemporary performance analytics frameworks that emphasize individualized load progression and continuous monitoring throughout the training cycle (12, 13). For volleyball coaches, incorporating ongoing mid-phase assessments and adjusting workloads based on neuromuscular responses may not only enhance performance outcomes but also reduce fatigue-related risks. Applying these principles ensures that physical improvements are effectively integrated with tactical preparation to optimize match performance.

## Limitations and future directions

The relatively small sample size (*n* = 24) reflects the fixed roster structure of collegiate volleyball teams, which typically consist of 12 male and 12 female athletes. Because each team maintains a stable athlete allocation for competition eligibility, increasing the number of participants beyond the available roster was not feasible. Although this limits generalizability to larger or elite populations, the homogeneous team structure provides a realistic representation of university-level training environments. Additionally, the motion-analysis subsample included only eight participants, which limits the generalizability of the kinematic findings. These results should therefore be interpreted as exploratory and supportive rather than conclusive. Future studies should employ larger kinematic samples or full-cohort biomechanical assessments to strengthen the interpretation of movement-pattern adaptations. Future studies involving multi-team or multi-institution datasets would help determine whether the observed adaptations are consistent across broader samples.

Although motion analysis was incorporated to confirm technical consistency during jump testing, the study did not collect detailed biomechanical variables such as joint angular displacement, take-off velocity, landing kinetics, or arm–leg coordination patterns. The primary purpose of the motion capture system was quality control rather than quantitative kinematic or kinetic evaluation. As a result, the biomechanical mechanisms underlying performance changes could not be examined directly. Future studies should integrate full biomechanical profiling (e.g., kinematics, kinetics, EMG, tendon stiffness) to better explain the determinants of training-induced adaptations.

Moreover, incorporating physiological assessments such as electromyography, rate of force development, or tendon stiffness would help clarify the mechanisms underlying training-induced performance improvements. Future research could also expand predictive modeling frameworks through machine learning and individualized load monitoring, enabling real-time athlete profiling and precision-based training management.

An additional limitation involves the lack of assessment regarding long-term retention of training-induced gains. Research suggests that neuromuscular adaptations from jump training can begin to decline within 3–6 weeks without continued stimulus, particularly among amateur or collegiate athletes (17). Therefore, incorporating low-volume, maintenance-phase plyometric training during the competitive season may be essential for sustaining the improvements observed in this study. Future longitudinal studies should examine whether the superior gains produced by plyometric training persist over extended training cycles and competitive periods.

## Short-form conclusion

In conclusion, plyometric training produced significantly greater gains in vertical jump performance than the Air Alert program among both male and female volleyball athletes. The findings emphasize the importance of progressive overload, adequate recovery, and individualized monitoring in maximizing explosive power development. Integrating predictive modeling into performance evaluation offers a valuable pathway for implementing data-driven and evidence-based training practices in volleyball.

## Data Availability

The original contributions presented in the study are included in the article/Supplementary Material, further inquiries can be directed to the corresponding author.
